# A New *TYR* Splice Donor Variant Causing Oculocutaneous Albinism Type I in Angus Cattle

**DOI:** 10.1002/age.70119

**Published:** 2026-05-08

**Authors:** Katie L. M. Eager, Phillip D. Carter, Lillian Brancalion, Cali E. Willet, Brendon A. O'Rourke, Imke Tammen

**Affiliations:** ^1^ Faculty of Science, Sydney School of Veterinary Science University of Sydney Camden New South Wales Australia; ^2^ Elizabeth Macarthur Agricultural Institute, NSW Department of Primary Industries and Regional Development Menangle New South Wales Australia; ^3^ Local Land Services, NSW Department of Primary Industries and Regional Development Casino New South Wales Australia; ^4^ Sydney Informatics Hub University of Sydney Camden New South Wales Australia

Variants in the *TYR* gene are known to cause autosomal recessive oculocutaneous albinism type I in multiple animal species (OMIA:000202) (Nicholas et al. 2025) and in humans (OMIM:606933) (Johns Hopkins University 2025). The *TYR* gene encodes tyrosinase, the rate‐limiting enzyme in the melanin biosynthesis pathway. Pathogenic variants in the *TYR* gene lead to a failure to produce pigmentation in the body and result in white hair, pale skin and light irises, as well as visual impairment due to disrupted eye development (Gronskov et al. 2007). In taurine cattle, two variants have been described: a one nucleotide insertion (omia.variant:589; c.925_926insC) in exon 2 resulting in a frameshift in Brown Swiss cattle (Schmutz et al. 2004) and a missense variant (omia.variant:1830; c.1283C>T; p.(P428L)) in exon 4 in Simmental cattle (Jacinto et al. 2025). We describe a novel splice donor variant in the *TYR* gene (omia.variant:1851; NM_181001.3:c.1184+1G>C; NC_037356.1:g.6389480C>G), as the likely causal variant for oculocutaneous albinism type I in two closely related Angus calves.

Over subsequent calving seasons, two affected animals were suspected to be blind, presenting with pink irises, constricted pupils, absent menace response and an unpigmented coat colour that covered the entire body (Figure S1). DNA extraction and whole genome sequencing of the two affected animals was performed as previously described (Eager et al. 2025). Variant filtering based on mode of inheritance and likely impact on protein function identified a common homozygous variant in the *TYR* gene of both affected animals that was absent in 21 control animals of different breeds. The identified *TYR* variant (NM_181001.3:c.1184+1G>C) affects the invariant +1G nucleotide of the canonical GT splice donor site, a position critical for normal splicing and for which substitutions are strongly predicted to disrupt splice‐site function. This variant was absent in the publicly available VCFs from the 1000 bulls genome project (Daetwyler et al. 2021), which includes over 100 Angus animals. Sanger sequencing using primers 5′‐GACTGAAGCGACTTAGCAGCA‐3′ and 5′‐GCAGATGCCTCTCAAAGCAG‐3′, confirmed that the parent verified common sire and both implicated dams were heterozygous carriers for the variant (Figure 1). Parentage analysis was performed as previously described (Eager et al. 2020) and confirmed reported inbreeding, with the bull mated to his dam to produce affected calf 1 and to his daughter to produce affected calf 2.

**FIGURE 1:**
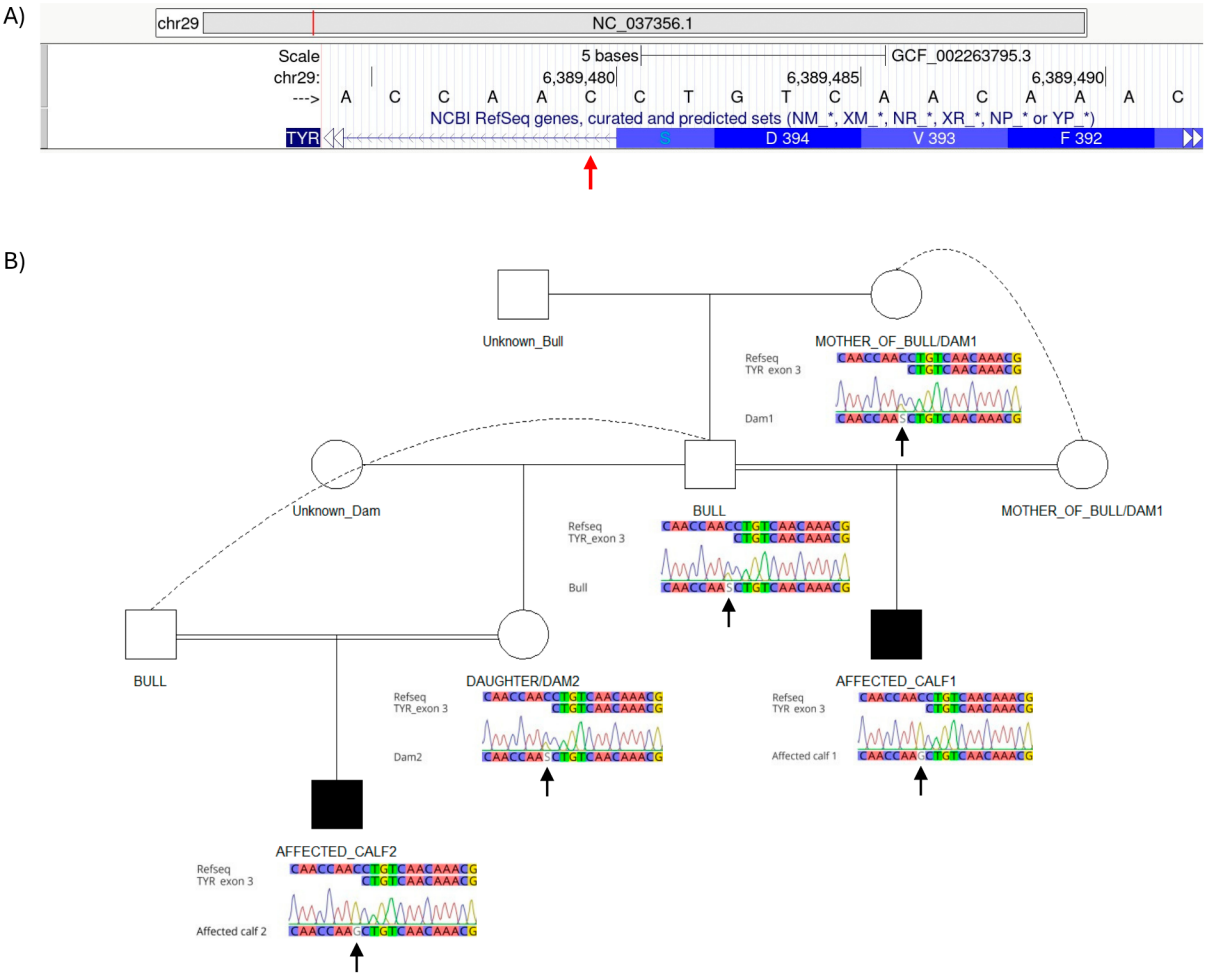
(A) Schematic of the partial bovine *TYR* gene from the UCSC genome browser showing exon–intron boundaries and predicted protein translation. The position of the *TYR* splice donor variant (NM_181001.3:c.1184+1G>C) is indicated by a red arrow. (B) Pedigree and Sanger sequencing chromatograms of the Angus animals used in this study. The *TYR* splice site variant is indicated by a black arrow.

Furthermore, Illumina 100 K SNP genotyping identified that the two affected animals shared a region of homozygosity that was absent in the parents and in other Angus animals, further supporting the *TYR* variant as being pathogenic (Figure S2). In addition, ClinVar has listed a C>G substitution at the corresponding position in the human ortholog as a pathogenic splicing variant in a patient with oculocutaneous albinism type 1A (National Center for Biotechnology Information 2025). Based on Animal Variant Classification Guidelines (AVCG) criteria (Boeykens et al. 2024), the variant is classified as pathogenic, fulfilling PVS1 (canonical splice site), PS5 (presence in multiple affected individuals), and PP1 (cross‐species support), with no evidence supporting a benign classification. We conclude that this variant is likely to adversely affect *TYR* function by disrupting a donor splice site, potentially resulting in exon skipping or activation of a nearby cryptic site, consistent with the observed phenotype in these affected Angus cattle. Identification of this variant enables carrier testing and prevention of further cases in the Angus population. Further work is recommended to characterise the specific functional impact of this variant in *TYR*, including gene expression and splicing analyses across genotypes from both normal and affected animals.

## Funding

This work was supported by the Anstee Hub for Inherited Diseases in Animals and NSW Department of Primary Industries and Regional Development.

## Ethics Statement

Samples from the affected herd and the control animals used in this study were collected as part of research projects approved by the University of Sydney Animal Ethics Committee (Project Numbers: 2016/998 and 2020/1720).

## Conflicts of Interest

The authors declare no conflicts of interest.

## Supporting information


**Figure S1:** Close‐up images of affected calf 1 (A) and affected calf 2 (B), showing pink irises and constricted pupils, together with an albino coat phenotype.

**Figure S2:** Region of homozygosity (ROH) analysis, where coloured lines represent runs of homozygosity in each animal. A shared ROH of > 10 Mbps and encompassing the *TYR* locus (AC_000186.1:6340841–6473276, indicated by a black arrow) was present in both affected animals. This ROH is absent in the Angus control animal and in all three parents (the two dams not shown as no ROH was detected on chromosome 29).

## Data Availability

The variant data for this study have been deposited in the European Variation Archive (EVA) at EMBL‐EBI under accession number PRJEB80029 (https://www.ebi.ac.uk/eva/?eva‐study=PRJEB80029).
