# Supplementary figures and images for: A New TYR Splice Donor Variant Causing Oculocutaneous Albinism Type I in Angus Cattle
Source: Anim Genet. 2026 May 8;57:e70119. doi: 10.1002/age.70119 (PMC13155181; doi:10.1002/age.70119)

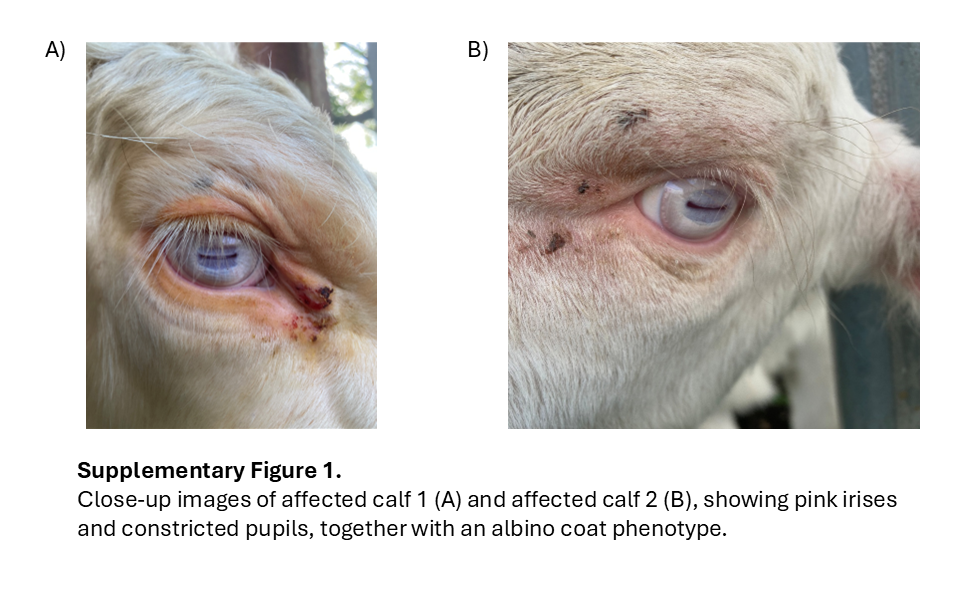

Supplement: Supplementary file 1 — Figure S1: Close‐up images of affected calf 1 (A) and affected calf 2 (B), showing pink irises and constricted pupils, together with an albino coat phenotype. [file AGE-57-0-s001.png]

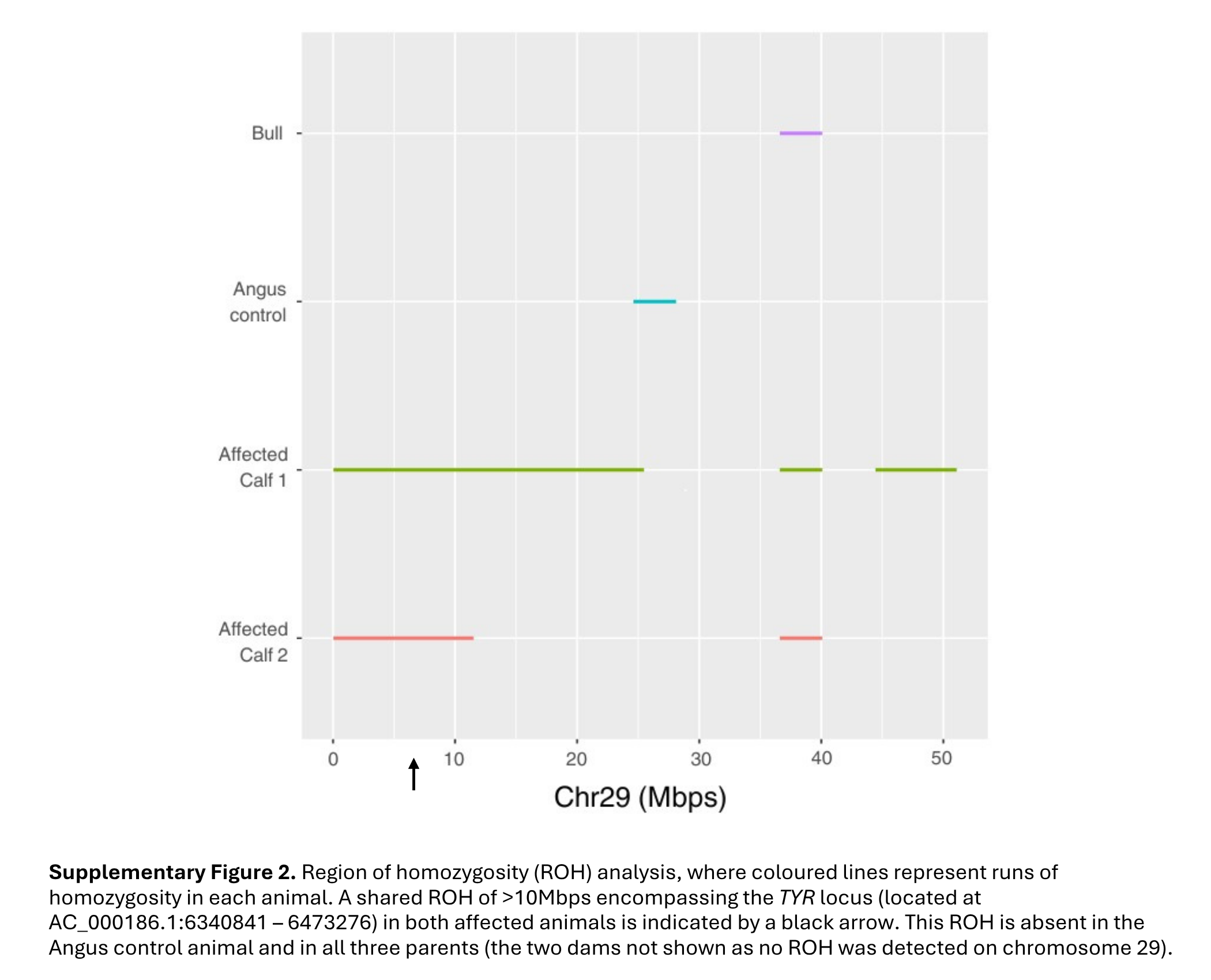

Supplement: Supplementary file 2 — Figure S2: Region of homozygosity (ROH) analysis, where coloured lines represent runs of homozygosity in each animal. A shared ROH of > 10 Mbps and encompassing the TYR locus (AC_000186.1:6340841–6473276, indicated by a black arrow) was present in both affected animals. This ROH is absent in the Angus control animal and in all three parents (the two dams not shown as no ROH was detected on chromosome 29). [file AGE-57-0-s002.png]
